# Supplementary material for: Rapid turnover of life-cycle-related genes in the brown algae
Source: Genome Biol. 2019 Feb 14;20:35. doi: 10.1186/s13059-019-1630-6 (PMC6374913; doi:10.1186/s13059-019-1630-6)
Supplement: Supplementary file 3 — Protein alignments, corresponding Gblocks html file and CDS sequences for the lineage conserved gametophyte-biased genes. (ZIP 2109 kb) [file 13059_2019_1630_MOESM3_ESM.zip › Con_GA_alignments/Ec-08_005500.aa_ali.fasta-gb.htm]

Ec-08\_005500.aa.fasta.trans.aa\_ali.fasta


## Gblocks 0.91b Results

Processed file: **Ec-08\_005500.aa.fasta.trans.aa\_ali.fasta**  
Number of sequences: **2**  
Alignment assumed to be: **Protein**  
New number of positions: **166** (selected positions are underlined in blue)

```
                         10        20        30        40        50        60
                 =========+=========+=========+=========+=========+=========+
Ec-08_005500     MSLDHESSLPKCLAGLSAHLQAEKEVIAVVRAIEDQAAEAVRDLALAELELPAGAAAVTA
MSTRG.14319.1    ------------------------------------------------------------
                                                                             


                         70        80        90       100       110       120
                 =========+=========+=========+=========+=========+=========+
Ec-08_005500     KLVPLLQWNKPSYDQASEQSPPGCLEPEPEPIPPSIDNWARGVVPCKNVSWVPGKLPSPS
MSTRG.14319.1    ------------------------------------------------------------
                                                                             


                        130       140       150       160       170       180
                 =========+=========+=========+=========+=========+=========+
Ec-08_005500     PRGDQEEEEPALRGGEEQDSSKMPVSNDHAGRRESKDKDATSRAAKYGADCNAARNRDDD
MSTRG.14319.1    ------------------------------------------------------------
                                                                             


                        190       200       210       220       230       240
                 =========+=========+=========+=========+=========+=========+
Ec-08_005500     DGRAWGGGNEEGEDIGLLSLFSEEGHGLGDEKLAGAGGIGDAIGRGRQHEERAFLESLRR
MSTRG.14319.1    ------------------------------------------------------------
                                                                             


                        250       260       270       280       290       300
                 =========+=========+=========+=========+=========+=========+
Ec-08_005500     REEARIQEKAQARERLERIDKENRAKEEKMKKELKGRAWVWGPNGEVIILNVVPPEKIPA
MSTRG.14319.1    RD----------------------------------------------IFTVVLRDKITP
                                                                 ############


                        310       320       330       340       350       360
                 =========+=========+=========+=========+=========+=========+
Ec-08_005500     RVMPRIGVRDRSADGGGSDVTGNTSPLRGGPHTKVAPAPTREGGDSKASKGGQVGSGNND
MSTRG.14319.1    KLFP--------------------------------------------------------
                 ####                                                        


                        370       380       390       400       410       420
                 =========+=========+=========+=========+=========+=========+
Ec-08_005500     TDGGGGSGGGGEGERRAKDNGGRGRRTKRNDGAAAARRRKGSRSERFFEMSATTQPPLVD
MSTRG.14319.1    ------------------------------------------------------------
                                                                             


                        430       440       450       460       470       480
                 =========+=========+=========+=========+=========+=========+
Ec-08_005500     TLDLQAGVCVQQGGDEKQGPAFEEDHKHPPRRDLLPRGPLPREPSLARAPTPVSIGDATS
MSTRG.14319.1    -------------------------------HDVLP--PYPRAPS---------------
                                                                             


                        490       500       510       520       530       540
                 =========+=========+=========+=========+=========+=========+
Ec-08_005500     VSAREGRSLDPPRGRRPSHGDPLSPPATHPLRSASIGSGSGRRARHHRVSSNRDSVVNRV
MSTRG.14319.1    --------------RGLVAGDPLSPPATFPLRSASIPAG---RGDNEHPSPGRNRLMDGV
                               #########################   ##################


                        550       560       570       580       590       600
                 =========+=========+=========+=========+=========+=========+
Ec-08_005500     KTLDPLSGGKRIEAG--GTGARGGGGVGHHRGRRLVVPGGAEPEGFGVGSDDDAEDDPHL
MSTRG.14319.1    KTLDPLSGATRLEPSGVGSGAAGRGEGGYHRGRRLSAPEGAEQEGFRVA--DDPEDDPHW
                 #############    ###############################   #########


                        610       620       630       640       650       660
                 =========+=========+=========+=========+=========+=========+
Ec-08_005500     RLFKEGGNIGGGGGGGGGFKPVPPPPLPRKRGSKQRRGVLAKPELRRPRDRGTQPPIPTV
MSTRG.14319.1    RLFKEGGNVSG---GAAGLKPVPPPALPKKRGPRHKRGALIKPEPRRPRDRGTRLPIS--
                 ###########   ###########################################   


                        670       680       690       700       710       720
                 =========+=========+=========+=========+=========+=========+
Ec-08_005500     TRKHLPPPPLGETMGHGLSPPRDRGLGETLGGGSDAGGPSLPGSYAARHGNTNNSDSKFK
MSTRG.14319.1    ------------------------------------------------------------
                                                                             


                        730       740       750       760       770       780
                 =========+=========+=========+=========+=========+=========+
Ec-08_005500     RGREGLAAHPADSGVGRKRKSGGGGAGGGSSESPHRMEGREKGGGKELPALPGQKKQQAF
MSTRG.14319.1    ------------------------------------------------------------
                                                                             


                        790       800       810       820       830
                 =========+=========+=========+=========+=========+=
Ec-08_005500     LPSSPGEGDGGDADGEDGGGRKARRRGSTSRMGWISSRRSKGTLQELFQTA
MSTRG.14319.1    ---------------------------------------------------
```

```
Parameters used
Minimum Number Of Sequences For A Conserved Position: 2
Minimum Number Of Sequences For A Flanking Position: 2
Maximum Number Of Contiguous Nonconserved Positions: 8
Minimum Length Of A Block: 10
Allowed Gap Positions: None
Use Similarity Matrices: Yes
```

```
Flank positions of the 6 selected block(s)
Flanks: [289  304]  [495  519]  [523  553]  [558  588]  [592  611]  [615  657]  

New number of positions in Ec-08_005500.aa.fasta.trans.aa_ali.fasta-gb:  166  (19% of the original 831 positions)
```
